# Modeling relationships between calving traits: a comparison between standard and recursive mixed models

**DOI:** 10.1186/1297-9686-42-1

**Published:** 2010-01-25

**Authors:** Evangelina López de Maturana, Gustavo de los Campos, Xiao-Lin Wu, Daniel Gianola, Kent A Weigel, Guilherme JM Rosa

**Affiliations:** 1 Department of Animal Sciences, University of Wisconsin, Madison, 53706, USA; 2 Departamento de Mejora Genética Animal, INIA, Carretera de La Coruña km 7.5, 28040 Madrid, Spain; 3 Department of Dairy Science, University of Wisconsin, Madison, 53706, USA; 4 Department of Biostatistics and Medical Informatics, University of Wisconsin, Madison, 53706, USA

## Abstract

**Background:**

The use of structural equation models for the analysis of recursive and simultaneous relationships between phenotypes has become more popular recently. The aim of this paper is to illustrate how these models can be applied in animal breeding to achieve parameterizations of different levels of complexity and, more specifically, to model phenotypic recursion between three calving traits: gestation length (GL), calving difficulty (CD) and stillbirth (SB). All recursive models considered here postulate heterogeneous recursive relationships between GL and liabilities to CD and SB, and between liability to CD and liability to SB, depending on categories of GL phenotype.

**Methods:**

Four models were compared in terms of goodness of fit and predictive ability: 1) standard mixed model (SMM), a model with unstructured (co)variance matrices; 2) recursive mixed model 1 (RMM1), assuming that residual correlations are due to the recursive relationships between phenotypes; 3) RMM2, assuming that correlations between residuals and contemporary groups are due to recursive relationships between phenotypes; and 4) RMM3, postulating that the correlations between genetic effects, contemporary groups and residuals are due to recursive relationships between phenotypes.

**Results:**

For all the RMM considered, the estimates of the structural coefficients were similar. Results revealed a nonlinear relationship between GL and the liabilities both to CD and to SB, and a linear relationship between the liabilities to CD and SB.

Differences in terms of goodness of fit and predictive ability of the models considered were negligible, suggesting that RMM3 is plausible.

**Conclusions:**

The applications examined in this study suggest the plausibility of a nonlinear recursive effect from GL onto CD and SB. Also, the fact that the most restrictive model RMM3, which assumes that the only cause of correlation is phenotypic recursion, performs as well as the others indicates that the phenotypic recursion may be an important cause of the observed patterns of genetic and environmental correlations.

## Background

Structural equation models (**SEM**) are well established and widely used in the social sciences. In quantitative genetics, these models were first suggested by Sewall Wright [1] but were ignored for many years. Recently, Gianola and Sorensen [2] suggested a model in which recursive and simultaneous relationships between phenotypes are considered in the context of a multiple-trait Gaussian model. This stimulated application of SEM in animal breeding and genetics (e.g., de los Campos et al. [3,4], Varona et al. [5], López de Maturana et al. [6], Wu et al. [7]). SEM can be used, for example, to explore potential relationships between variables of interest or to evaluate the plausibility of different hypotheses [8]. In addition, SEM facilitate comparisons between alternative nested path analysis models [9].

López de Maturana et al. [10] applied SEM to study relationships between three calving traits (gestation length (**GL**), calving difficulty (**CD**) and stillbirth (**SB**)). SEM were found useful for detecting heterogeneous correlations between residual, contemporary group, or genetic effects affecting GL and liabilities to CD and SB. However, a comparison between their model and nested models with different restrictions on relationships between variables has not been addressed yet.

The present work complements the study of López de Maturana et al. [10] by comparing, in terms of goodness of fit and predictive ability, a sequence of SEM with different restrictions on the (co)variance matrices among model parameters.

## Methods

### Data

The data consisted of a sample of primiparous US Holstein cows calving from 2000 to 2005 that were recorded as part of the National Association of Animal Breeders (Columbia, Mo) Calving Ease Program. After editing, the data set contained GL, CD and SB records from 90,393 cows, sired by 1,122 bulls, mated to 567 service sires, and distributed over 935 herd-calving year combinations, as described in López de Maturana et al. [10].

### Statistical model

The general specification of the model is given in López de Maturana et al. [10]. The model allows for recursive effects that change according to categories of GL (261-267 d, 268-273 d, 274-279 d, and 280-291 d). The observable phenotypes were ; for CD and SB threshold links were used; and the measurement models for these traits were,(1)

where (), and () denote liabilities and thresholds for CD (SB), respectively. For identification purposes, the first thresholds for CD () and SB () were set to 0 and the second threshold for CD () was set to 1. A multivariate normal model was assumed for .

The reduced-form equation for  was:(2)

In the above, *k *denotes the category of GL; **μ**_*i *_= **X**_*i*_b+**Z**_*i*(*h*)_**h**+**Z**_*i*(*s*)_**s**+**Z**_*i*(*mgs*)_**mgs; X**_*i*_**b **is the contribution to the linear predictor of systematic effects, including sex of calf (2 levels), age at first calving (4 levels), and year-season (12 levels); **Z**_*i*(*h*)_**h**, **Z**_*i*(*s*)_**s **and **Z**_*i*(*mgs*)_**mgs **represent the contributions of herd-year (935 levels), sire (567 levels with progeny), and maternal grandsire effects (1,122 levels with progeny), respectively; and **Λ**_*k *_is a 3 × 3 matrix defining recursive effects of the following form:(3)

where, *λ*_*CD*←*GL*(*k*)_, *λ*_*SB*←*GL*(*k*) _and *λ*_*SB*←*GL*(*k*) _describe rates of change of the liabilities to CD and SB with respect to GL, and of the liability to SB with respect to the liability to CD, respectively. As noted before, recursive coefficients were allowed to vary across categories of GL, *k *= {1, if  ≤ 267 d; 2, if 267 d < ≤ 273 d; 3, if 273 d < ≤ 279 d; 4, otherwise}, to account for non-linearity of the relationship between GL and the two calving traits. Model residuals, **ε**_*i*_, were assumed to be independent and identically distributed (IID) across animals, that is, , where **R**_0 _is a 3 × 3 residual (co)variance matrix, with its last diagonal entry (i.e., the residual variance of the liability to SB) restricted to 1 for identification purposes.

#### Prior distribution

The prior distribution was factorized as follows:(4)

where, **θ**_*k *_= (**Λ**_*k*_, **b**, **h**, **s**, **mgs**, **G**_0_, **H**_0_, **R**_0_, τ); **G**_0 _and **H**_0 _are (co)variance matrices of genetic, herd and residual effects, respectively; **λ**_*k *_is a vector containing the non-null recursive effects; and **τ **is the vector with the thresholds.

#### (Co)variance components

The reduced model (2) implies that the (co)variance matrices due to genetic, permanent environmental effects and model residuals are,(5)

With , , ,  and , where, for example,  is the between-sire variance for GL,  is the (co)variance between sire effects of GL and CD,  and  are the herd-year and residual variances for GL, and  and  are the herd-year and residual covariances between GL and CD, respectively. Additive direct and maternal genetic (co)variances were calculated according to Willham [11]:(6)

Where , , ,  are the variances of additive direct genetic effects, additive maternal genetic effects, sire, and maternal grandsire effects, respectively; *σ*_*dm *_and *σ*_*smgs *_are the covariances between additive direct and maternal genetic effects and between sire and maternal grandsire effects, respectively. The genetic (co)variances were computed following [12]:(7)

Without imposing further restrictions, the model described in (2) considering the recursive relationship is under-identified. Identification can be attained by imposing restrictions on dispersion, location parameters or on the matrix of recursive effects. For computational convenience and due to the difficulty to assure identification through the location parameters, only restrictions on dispersion or recursive parameters were considered. A sequence of models was obtained by changing the prior specifications for *p*(λ), *p*(**G**_0_), *p*(**H**_0_), and *p*(**R**_0_)

### Recursive mixed model 1 (RMM1)

This model assumes that the correlation between residuals in the reduced models, **Λ**^-1^_k_**ε**_i_, is solely a consequence of the phenotypic recursion. **R**_0 _is assumed to be diagonal, i.e., *p*(**R**_0_)is the product of two independent scaled inverted Chi-square distributions (for GL and CD, because  was set to 1 to ensure identification), and *p*(**G**_0_) and *p*(**H**_0_) are assumed to be distributed a priori as inverted Wishart distributions. The number of unknowns in the dispersion parameters and the matrix of recursive effects is 41: 6 in ,  and **H**_0_, 9 in , 2 in **R**_0_, and 3 in each **Λ**_*k*_.

### Recursive mixed model 2 (RMM2)

This model results from adding to RMM1 the restriction that **H**_0 _is also diagonal. This restriction implies that the correlations between residuals and between contemporary groups in the reduced model are exclusively due to recursive relationships. Thus, the number of parameters entering in [5] in RMM2 (38) is smaller than those entering in [5] in model RMM1 (number of parameters equal to 41). RMM2 is obtained by assigning an inverted Wishart distribution to **G**_0 _and independent scaled-inverted Chi-square distributions to the unknown diagonal elements of **H**_0 _and **R**_0_. Note that, as in RMM1,  is set to 1 to ensure identification.

### Recursive mixed model 3 (RMM3)

This model assumes that the only cause of correlations between any of the random effects in the reduced model is the phenotypic recursion. That is, , , , **H**_0 _and **R**_0 _are diagonal, and the priors for the unknown diagonal components are independent scaled-inverted Chi-square distributions. The number of unknowns in dispersion parameters and in the matrix of recursive effects is now 26.

### Standard mixed model (SMM)

This model is defined by setting and **Λ**_k _= **I**, and by treating **G**_0_, **H**_0_, and **R**_0 _as unstructured (co)variance matrices. As prior distributions, inverted Wishart distributions are assumed to **G**_0 _and **H**_0 _and a conditional inverted Wishart distribution to **R**_0 _(*p*(**R**_0_|| = 1)) (see [13] for details). The sum of unknowns in the (co)variance matrices is 32 (6 in **H**_0_, 5 in **R**_0 _and 21 in **G**_0_); there are no recursive parameters in this model.

#### Implementation

With the a priori assumptions described above, the fully conditional distributions of all unknowns in all models have closed forms, and draws from the posterior distribution can be obtained via Gibbs sampling. The SirBayes software [7] was used to implement the models. The length of the chain and the burn-in period were assessed by visual examination of trace plots of posterior samples of selected parameters; additional diagnostic checks were employed. After a preliminary analysis, it was decided to run 5 independent chains, each consisting of 10,000 iterations. In each chain, the first 1,000 iterations were discarded as burn-in, and one of every 10 successive samples was retained. Thus, 4,500 samples were used to infer the posterior distributions of unknown parameters. Features of the marginal posterior distributions of interest, the convergence analysis, and estimates of Monte Carlo error, were obtained using the BOA software http://www.public-health.uiowa.edu/boa.

### Model comparison

The performance of the SMM and the three RMM considered was investigated in terms of both goodness of fit and predictive ability, under the consideration that a model that fits current data very well may fail to provide accurate predictions of future (independent) observations [14].

The mean squared error of a calving trait phenotype, , and Pearson's correlation between fitted and observed data, , were evaluated at the posterior means of the unknowns (), to assess goodness of fit.

Predictive ability was assessed with MSE and Pearson's correlation, using a 3-fold cross-validation (CV) procedure. The full data set was randomly partitioned into three disjoint subsets, each with approximately one-third of the records. The CV procedure used two of the three subsets for model fitting and prediction (i.e., the training set), and predictive ability was evaluated in the remaining subset (i.e., the testing set). MSE and Pearson's correlation were computed as before, but in this case by concatenating results from the three cross-validation sets.

The predicted or fitted values for CD and SB were computed as:(8)

where the probability that observation *i *falls in category *c *was calculated as:(9)

Above, **Φ**(·) is the cumulative distribution function of a standard normal variate; **τ**_c _is the assumed (or estimated) value of the appropriate threshold for CD and SB, and  is the posterior mean of the liability to CD or SB for individual *i*.

## Results and Discussion

Small Monte Carlo errors (~10^-2^-10^-4^) were obtained for all the parameters that were estimated in each model; this suggests that convergence was achieved, and that a sufficient number of Gibbs samples was used.

### Structural coefficients

Posterior means (standard deviations) of structural coefficients obtained from the analyses of the recursive models (RMM1, RMM2 and RMM3) are shown in Table 1. Similar estimates were found in the three models. For gestations within 261-267 d, an extra day of gestation did not increase CD. Calving problems did increase for the remaining groups of GL, because the rates of changes were positive, and the HPD_95% _(Highest Posterior Density at 95% of probability) region did not include 0. Different rates of change of the liability to SB for different categories of GL were found as a consequence of direct (*λ*_*SB*←*GL*_) and indirect recursive effects (*λ*_*CD*←*GL *_× *λ*_*SB*←*CD*_): the liability to SB was expected to decrease in the two first categories (261-273 d), not to change in the third category (274-279 d) and to increase in the fourth category (280-291 d). Positive estimates (similar across categories of GL) were found for the effect of the liability to CD on the liability to SB, indicating that cows that are more likely to suffer calving difficulty are more likely to have stillborn calves. More details regarding the recursive relationships between GL, CD and SB can be found in López de Maturana et al. [10].

**Table 1:** Posterior mean (standard deviation) of structural coefficients for calving traits from the recursive mixed models

**Structural coefficients**	**Model^a^**	**Category of GL**			
		**261-267 d**	**268-273 d**	**274-279 d**	**280-291 d**
				
*λ*_*CD*←*GL *_(l. u.^b^/1 d GL)	RMM1	0.005 (0.005)	0.020** (0.003)	0.032** (0.005)	0.040** (0.003)
	RMM2	0.006 (0.005)	0.020** (0.003)	0.032** (0.005)	0.040** (0.003)
	RMM3	0.005 (0.005)	0.021** (0.003)	0.033** (0.005)	0.041** (0.003)
Overall effect of GL on SB (l. u./1 d GL)^c^	RMM1	-0.044** (0.006)	-0.021** (0.004)	-0.008 (0.006)	0.024** (0.003)
	RMM2	-0.044** (0.0062)	-0.021** (0.0038)	-0.008 (0.0057)	0.025** (0.0031)
	RMM3	-0.044** (0.006)	-0.021** (0.004)	-0.008 (0.006)	0.025** (0.003)
*λ*_*SB*←*CD *_(l. u./l. u. CD)	RMM1	0.339** (0.023)	0.331** (0.011)	0.330** (0.007)	0.3311** (0.007)
	RMM2	0.327** (0.023)	0.319** (0.010)	0.317** (0.007)	0.318** (0.007)
	RMM3	0.330** (0.003)	0.321** (0.011)	0.319** (0.007)	0.320** (0.007)

** 99% highest posterior density region, HPD_99%_, does not include 0; ^a^RMM1: recursive mixed model (RMM) assuming that the relationship between residuals is due to the recursive relationships between the gestation length (GL) phenotype and the liabilities to calving difficulty (CD) and stillbirth (SB); RMM2: RMM assuming that the relationships both between residuals and between herd-years are due to the recursive relationships between the phenotype of GL and the liabilities to CD and SB; RMM3: recursive mixed model assuming that phenotypic correlations of the system are uniquely caused by the recursiveness; ^b ^l. u.: liability units; ^c^The overall recursive effect of GL on liability to SB is the sum of the direct and indirect recursive effects, *λ*_*SB*←*GL *_+ *λ*_*CD*←*GL *_× *λ*_*SB*←*CD*_

### Genetic parameters

Additional file 1, Table S1 shows the posterior means (standard deviations) of direct and maternal heritabilities of GL and liabilities to CD and SB for each model. Posterior distributions of direct and maternal heritabilities for the three calving traits were similar across categories of GL and between models (RMM1, RMM2 and RMM3) and were also similar to their counterparts from the SMM. The posterior mean of direct heritability of GL was higher than that for maternal heritability (0.39 vs. 0.08-0.07); corresponding estimates for CD (0.08-0.10 vs. 0.07-0.08) and SB (0.05-0.08 vs. 0.08-0.11) were smaller than those for direct heritability and similar between them. Heritability estimates were within the range of values reported in previous studies [15-17]; estimates for CD and SB were higher than those used in routine genetic evaluations of CD and SB in US Holsteins, except for the direct heritability of CD [18,19].

Features of the posterior distributions of genetic correlations in the four categories of GL from the SMM and RMM models are shown in Additional file 1, Tables S2, S3, S4 and S5. In general, estimates of genetic correlations obtained from the SMM were within the ranges of values obtained for each category of GL from the RMM analyses. All of the recursive models evaluated in this study detected a heterogeneous correlation between direct and maternal effects of GL and between direct and maternal liabilities to CD and SB, as expected. Similar estimates were found in the analyses of RMM1 and RMM2. Regarding the correlation between direct effects of GL and CD, positive posterior means were obtained from both SMM and RMM by category of GL. For all categories of GL, RMM3 gave lower estimates than the other models, due to restrictions placed on **G**_0_. Similarly, positive estimates (although slightly lower) were found between maternal effects of GL and CD. Slightly stronger correlations between direct effects of GL and SB were found using RMM3, compared with those using RMM1 or RMM2, for all categories of GL. Relatively high, positive, and similar estimates were obtained for the genetic correlation between direct effects for CD and SB in each of the four categories of GL, with lower estimates from RMM3. A similar pattern, although with slightly lower estimates, was found for the genetic correlation between the maternal effects of CD and SB.

Similar posterior means of the genetic correlation between direct and maternal effects for the same trait were found in SMM and RMM, and across categories of GL: moderately negative for GL and SB, and close to 0 for CD.

The 90% highest posterior density intervals for genetic correlations between direct and maternal effects for different traits obtained with RMM included 0 or had an almost null posterior mean, and were similar to their counterparts from the SMM. This suggests that effects of genes controlling direct effects for one calving trait are not associated with those controlling maternal effects for another calving trait, and vice versa.

The estimates of previously genetic correlations were within the range of values reported in the literature [15-17].

Additional file 1, Table S6 shows the posterior means of correlations between contemporary groups and between residuals. Almost null estimates of the correlation between contemporary groups of GL and CD were found in SMM and RMM for all categories of GL. Regarding GL and SB, small positive estimates were obtained from the analyses of SMM and RMM1. Results from RMM1 suggest that the correlation changes across categories of GL. Estimates from the other recursive models (RMM2 and RMM3) also suggested that the correlation changes across categories of GL, including a modification of sign: slightly negative in the first two categories of GL (-0.10 and -0.05, respectively), nil in the third, and slightly positive in the fourth (0.06). Posterior means of the correlation between herd-year effects of CD and SB were nil in the analyses of models SMM and RMM1; however, those from models RMM2 and RMM3 were moderate and positive (0.54). Differences in sign and magnitude between estimates were a consequence of the different assumptions regarding the covariances between herd-year effects in SMM and RMM1 *versus *those in RMM2 and RMM3.

The RMM detected heterogeneous correlations between residuals of GL and both CD and SB that were solely due to the recursive relationship between GL and liabilities to CD and SB residuals. Estimates from SMM were in the interval of values from RMM. Similarly, positive and moderate correlations between residuals of CD and SB were found in all RMM models (0.38-0.40), whereas the estimate from SMM was much lower (0.09).

### Model comparison

Among the variety of model comparison methods, MSE and Pearson's correlation between observed and estimated/predicted phenotypes were chosen based on their ease of interpretation and weaker dependence on priors' choice. Mean squared error is a measurement related to the bias-variance trade-off of a model, either for fitting or predictive ability, whereas Pearson's correlation indicates the accuracy of estimations/predictions. The use of these criteria provides information on the model performance for each analyzed trait, but they lack an overall measure of the multivariate model performance. Bayes Factor or DIC could be alternative model selection criteria to provide such information. However, due to their disadvantages, which will be briefly described below, we have discarded them in favor of MSE and Pearson's correlation. Bayes Factor is based on marginal likelihood, and therefore provides a measure of model goodness of fit. This criterion indicates whether the data increased or decreased the odds of model *i *relative to model *j *[14]. However, it depends on prior input, and this dependence does not decrease as sample size increases, unlike parameter's estimation based on posterior distributions [20]. In addition, BF does not indicate which hypothesis is the most probable, but it shows which hypothesis would make the sample more probable, if the hypothesis is true and not otherwise. Regarding DIC, it makes a compromise between goodness of fit and model complexity, and in some contexts, it can agree with measures of predictive ability. However, this is not always the case. Additionally, DIC is based on an approximation that may not be appropriate in the class of non-linear models considered here.

#### Goodness of fit

**Figure 1:**
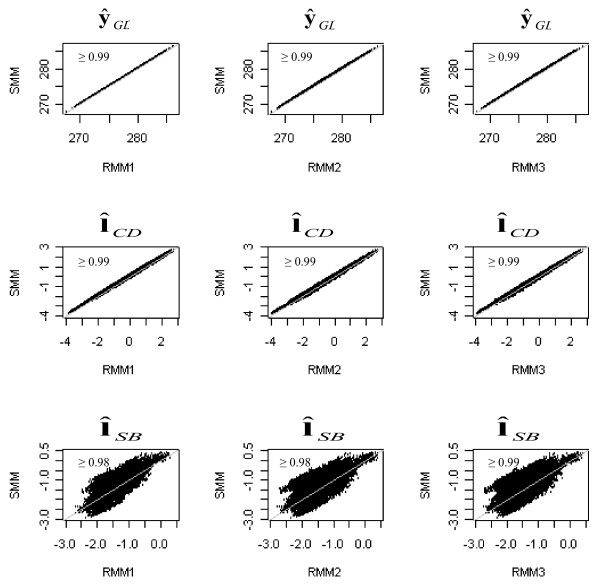
Plots and Pearson's correlations between the posterior means of expected gestation length () and of expected liabilities to calving difficulty () and stillbirth () obtained with standard mixed models SMM versus those obtained with recursive mixed models (RMM)

Figure 1 displays scatter plots of the expected GL () and the posterior mean of expected liabilities to CD and SB ( and ) obtained with SMM against those obtained with RMM. As expected, similar posterior means of  were obtained from SMM and RMM (Pearson's correlation near 1), because the model for GL is not affected by the structure imposed in recursive models. The correlation between the posterior means of liability to CD from the SMM and each of the RMM were also close to 1, with very slight differences between them. However, a weaker association was found between the posterior means of liabilities to SB estimated with SMM and each of the RMM (Pearson's correlations around 0.69-0.70).

**Figure 2:**
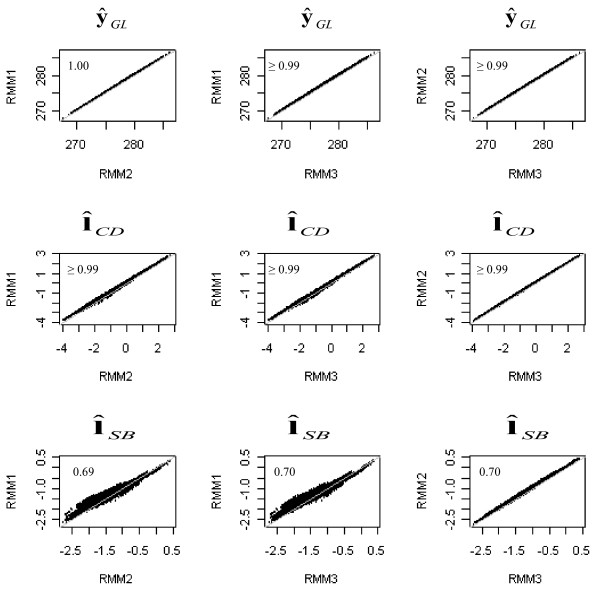
Plots and Pearson's correlations between the posterior means of expected gestation length () and of expected liabilities to calving difficulty () and stillbirth () obtained with the recursive mixed models (RMM)

Figure 2 shows the plots of the posterior mean of the expected GL and liabilities to CD and SB obtained with one of the RMM against those of the remaining recursive models. Again, the posterior means of the estimated phenotype of GL and the liabilities to CD obtained from the different RMM were similar, with correlations of ≥ 0.99. Estimated liabilities from RMM2 and RMM3 were also similar, with a correlation of 0.99. Correlations between estimates from RMM1 and RMM2 and estimates from RMM1 and RMM3 were slightly lower (0.98).

Table 2 shows the average MSE and Pearson's correlation between fitted and observed phenotypes of GL, CD and SB, by model. The goodness of fit measures did not change across models, with differences at the third decimal place.

**Table 2:** Goodness of fit criteria for standard (SMM) and recursive (RMM) mixed models

	Comparison criteria	Model^a,b^			
		SMM	RMM1	RMM2	RMM3
GL					
	Mean squared error	18.717	18.717	18.716	**18.715**
	Pearson's correlation	0.465	0.465	0.465	**0.465**
CD					
	Mean squared error	**0.788**	0.791	0.791	0.791
	Pearson's correlation	**0.487**	0.485	0.486	0.486
SB					
	Mean squared error	**0.108**	0.109	0.109	0.109
	Pearson's correlation	**0.246**	0.243	0.244	0.243

^a ^Boldface numbers indicate the best performance in goodness of fit, by criterion of comparison; ^b ^RMM1: recursive mixed model assuming that the relationship between residuals is due to the recursive relationships between the gestation length (GL) phenotype and the liabilities to calving difficulty (CD) and stillbirth (SB); RMM2: RMM assuming that the relationships both between residuals and between herd-years are due to the recursive relationships between the phenotype of GL and the liabilities to CD and SB; RMM3: recursive mixed model assuming that phenotypic correlations of the system are uniquely caused by the recursiveness

The differences observed between the posterior mean liabilities to SB from SMM and those from RMM (see Figure 1) did not occur when the goodness of fit of these models was evaluated in terms of MSE and Pearson's correlation between predicted and observed SB score.

#### Predictive ability

Table 3 presents the average MSE and Pearson's correlation between predicted and observed phenotypes of GL, CD and SB, by model. Both RMM and SMM had similar predictive abilities of GL and CD. Regarding SB, the model with best predictive ability was RMM1, with a 2.2% higher Pearson's correlation than other RMM. The differences in predictive ability among RMM were very small.

**Table 3:** Predictive ability of standard (SMM) and recursive mixed models from the analyses of cross-validation subsets

	Comparison criteria	Model^a,b^			
		SMM	RMM1	RMM2	RMM3
GL					
	Average mean squared error	19.559	19.559	**19.558**	**19.558**
	Pearson's correlation	0.424	0.424	**0.424**	**0.424**
CD					
	Average mean squared error	0.824	**0.823**	0.824	**0.823**
	Pearson's correlation	0.448	**0.450**	0.449	**0.450**
SB					
	Average mean squared error	0.111	**0.111**	0.111	0.111
	Pearson's correlation	0.150	**0.172**	0.170	0.170

^a^Boldface numbers indicate the best performance by criterion of comparison; ^b^RMM1: recursive mixed model (RMM) assuming that the relationship between residuals is due to the recursive relationships between the gestation length (GL) phenotype and the liabilities to calving difficulty (CD) and stillbirth (SB); RMM2: RMM assuming that the relationships both between residuals and between herd-years are due to the recursive relationships between the phenotype of GL and the liabilities to CD and SB; RMM3: recursive mixed model assuming that phenotypic correlations of the system are uniquely caused by the recursiveness

The negligible differences in terms of goodness of fit and predictive ability between models might be explained by the small differences in estimated genetic correlations between SMM (off diagonals of ,  and ) and RMM (off diagonals of ). The larger differences observed in correlations between contemporary groups for GL and liability to SB and between liabilities to CD and SB, as well as their counterparts between residual effects from SMM and RMM, were not reflected in goodness of fit and predictive ability. Thus, a very restrictive model (RMM3, with 26 parameters) provided similar fit and predictive ability as less parsimonious models.

## Conclusions

This paper illustrates how SEM can be used to achieve parameterizations with different levels of complexity that represent different genetic models. For example, recursive relationships can be used to generate models in which the genetic parameters are themselves subject to genetic variation.

The applications examined in this study suggest the plausibility of a recursive effect from GL onto CD and SB. Also, as reported in previous studies, this relationship is not linear. The fact that the most restrictive model (RMM3), which assumes that the only cause of correlation is phenotypic recursion, performs as well as the others indicates that the recursion may be an important cause of the observed genetic and environmental correlations.

## Competing interests

The authors declare that they have no competing interests.

## Authors' contributions

ELM conceived, carried out the study and wrote the manuscript; GC conceived, supervised the study and wrote the manuscript; XLW developed the software and revised the manuscript; DG, KW and GR helped to coordinate the study, provided critical insights and revised the manuscript.

## Supplementary Material

Additional file 1 Table S1 - Posterior means (standard deviations) of direct (d) and maternal (m) heritabilities of calving traits. Table S2 - Posterior means (standard deviations) of the genetic correlations, for gestations within 261-267 d. Table S3 - Posterior means (standard deviations) of the genetic correlations, for gestations within 268-273 d. Table S4 - Posterior means (standard deviations) of the genetic correlations, for gestations within 274-279 d. Table S5 - Posterior means (standard deviations) of the genetic correlations, for gestations within 280-291 d. Table S6 - Posterior means (standard deviations) of correlations between contemporary (h) groups and residual (e) effects. Click here for file

## References

[B1] Wright S The method of path coefficients The Annals of Mathematical Statistics 1934 5 3 161 215 10.1214/aoms/1177732676

[B2] Gianola D Sorensen D Quantitative genetic models for describing simultaneous and recursive relationships between phenotypes Genetics 2004 167 1407 1424 10.1534/genetics.103.025734 15280252 PMC1470962

[B3] de los Campos G Gianola D Boettcher P Moroni P A structural equation model for describing relationships between somatic cell score and milk yield in dairy goats J Anim Sci 2006 84 2934 2941 10.2527/jas.2006-016 17032786

[B4] de los Campos G Gianola D Heringstad B A structural equation model for describing relationships between somatic cell score and milk yield in first-lactation dairy cows J Dairy Sci 2006 89 4445 4455 17033034 10.3168/jds.S0022-0302(06)72493-6

[B5] Varona L Sorensen D Thompson R Analysis of litter size and average litter weight in pigs using a recursive model Genetics 2007 177 1791 1799 10.1534/genetics.107.077818 17720909 PMC2147959

[B6] López de Maturana E Legarra A Varona L Ugarte E Analysis of fertility and dystocia in Holsteins using recursive models to handle censored and categorical data J Dairy Sci 2007 90 2012 2024 10.3168/jds.2005-442 17369243

[B7] Wu X-L Heringstad B Chang YM de los Campos G Gianola D Inferring relationships between somatic cell score and milk yield using simultaneous and recursive models J Dairy Sci 2007 90 3508 3521 10.3168/jds.2006-762 17582135

[B8] Hershberger SL Marcoulides GA Parramore MM Structural equation modeling. Applications in ecological and evolutionary biology 2003 Cambrigde, UK: The press sindicate of the University of Cambridge

[B9] Bollen KA Structural equations with latent variables. NewYork 1989

[B10] López de Maturana E Wu X-L Gianola D Weigel KA Rosa GJM Exploring biological relationships between calving traits in primiparous cattle with a Bayesian recursive model Genetics 2009 181 277 287 10.1534/genetics.108.094888 18984571 PMC2621175

[B11] Willham RL The role of maternal effects in animal breeding: III. Biometrical aspects of maternal effects in animal breeding J Anim Sci 1972 35 1288 1292 4567216 10.2527/jas1972.3561288x

[B12] Kriese LA Bertrand JK Benyshek LL Age adjustment factors, heritabilities and genetic correlations for scrotal circumference and related growth traits in Hereford and Brangus bulls J Anim Sci 1991 69 478 489 2016177 10.2527/1991.692478x

[B13] Korsgaard IR Andersen AH Sorensen D A useful reparameterisation to obtain samples from conditional inverse Wishart distributions Genet Sel Evol 1999 31 177 181 10.1186/1297-9686-31-2-177

[B14] Sorensen DA Gianola D Likelihood, Bayesian, and MCMC Methods in Quantitative Genetics 2002 Springer-Verlag New York, Inc., 175 Fifth Avenue, New York

[B15] Heringstad B Chang YM Svendsen M Gianola D Genetic analysis of calving difficulty and stillbirth in Norwegian Red cows J Dairy Sci 2007 90 3500 3507 10.3168/jds.2006-792 17582134

[B16] Hansen M Lund MS Pedersen J Christensen LG Gestation length in Danish Holsteins has weak genetic associations with stillbirth, calving difficulty, and calf size Livest Prod Sci 2004 91 23 33 10.1016/j.livprodsci.2004.06.007

[B17] Steinbock L Näsholm A Berglund B Johansson K Philipsson J Genetic effects on stillbirth and calving difficulty in Swedish Holsteins at first and second calving J Dairy Sci 2003 86 2228 2235 12836960 10.3168/jds.S0022-0302(03)73813-2

[B18] Wiggans GR Misztal I Van Tassell CP Calving ease (co)variance conponents for a sire-maternal grandsire threshold model J Dairy Sci 2003 86 1845 1848 12778596 10.3168/jds.S0022-0302(03)73771-0

[B19] Cole JB Wiggans GR VanRaden PM Miller RH Stillbirth (co)variance components for a sire-maternal grandsire threshold model and development of a calving ability index for sire selection J Dairy Sci 2007 90 2489 2496 10.3168/jds.2006-436 17430953

[B20] Berger JO Pericchi LR The Intrinsic Bayes Factor for Model Selection and Prediction J Am Stat Assoc 1996 91 109 122 10.2307/2291387

