# Supplementary material for: Modeling relationships between calving traits: a comparison between standard and recursive mixed models
Source: Genet Sel Evol. 2010 Jan 25;42(1):1. doi: 10.1186/1297-9686-42-1 (PMC2830933; doi:10.1186/1297-9686-42-1)
Supplement: Additional file 1 — Table S1 - Posterior means (standard deviations) of direct (d) and maternal (m) heritabilities of calving traits. Table S2 - Posterior means (standard deviations) of the genetic correlations, for gestations within 261-267 d. Table S3 - Posterior means (standard deviations) of the genetic correlations, for gestations within 268-273 d. Table S4 - Posterior means (standard deviations) of the genetic correlations, for gestations within 274-279 d. Table S5 - Posterior means (standard deviations) of the genetic correlations, for gestations within 280-291 d. Table S6 - Posterior means (standard deviations) of correlations between contemporary (h) groups and residual (e) effects. [file 1297-9686-42-1-S1.DOC]

**Additional Material files**

**Table 1 - Posterior means (standard deviations) of direct (d) and maternal (m) heritabilities of calving traits**

| Heritabilities | Model ^a^ | Category of GL | | | |
| --- | --- | --- | --- | --- | --- |
|  |  | 261-267 d | 268-273 d | 274-279 d | 280-291 d |
| $h_{d_{GL}}^{2}$ | SMM | 0.39 (0.03) | 0.39 (0.03) | 0.39 (0.03) | 0.39 (0.03) |
|  | RMM1 | 0.39 (0.03) | 0.39 (0.03) | 0.39 (0.03) | 0.39 (0.03) |
|  | RMM2 | 0.39 (0.03) | 0.39 (0.03) | 0.39 (0.03) | 0.39 (0.03) |
|  | RMM3 | 0.39 (0.03) | 0.39 (0.03) | 0.39 (0.03) | 0.39 (0.03) |
| $h_{d_{CD}}^{2}$ | SMM | 0.10 (0.01) | 0.10 (0.01) | 0.10 (0.01) | 0.10 (0.01) |
|  | RMM1 | 0.08 (0.01) | 0.08 (0.01) | 0.08 (0.01) | 0.08 (0.01) |
|  | RMM2 | 0.08 (0.01) | 0.08 (0.01) | 0.08 (0.01) | 0.09 (0.01) |
|  | RMM3 | 0.08 (0.01) | 0.08 (0.01) | 0.08 (0.01) | 0.08 (0.01) |
| $h_{d_{SB}}^{2}$ | SMM | 0.08 (0.01) | 0.08 (0.01) | 0.08 (0.01) | 0.08 (0.01) |
|  | RMM1 | 0.07 (0.01) | 0.06 (0.01) | 0.06 (0.01) | 0.07 (0.01) |
|  | RMM2 | 0.06 (0.01) | 0.06 (0.01) | 0.06 (0.01) | 0.07 (0.01) |
|  | RMM3 | 0.06 (0.01) | 0.05 (0.01) | 0.05 (0.01) | 0.05 (0.01) |
| $h_{m_{GL}}^{2}$ | SMM | 0.08 (0.01) | 0.08 (0.01) | 0.08 (0.01) | 0.08 (0.01) |
|  | RMM1 | 0.08 (0.01) | 0.08 (0.01) | 0.08 (0.01) | 0.08 (0.01) |
|  | RMM2 | 0.08 (0.01) | 0.08 (0.01) | 0.08 (0.01) | 0.08 (0.01) |
|  | RMM3 | 0.07 (0.01) | 0.07 (0.01) | 0.07 (0.01) | 0.07 (0.01) |
| $h_{m_{CD}}^{2}$ | SMM | 0.08 (0.01) | 0.08 (0.01) | 0.08 (0.01) | 0.08 (0.01) |
|  | RMM1 | 0.07 (0.01) | 0.07 (0.01) | 0.07 (0.01) | 0.07 (0.01) |
|  | RMM2 | 0.07 (0.01) | 0.07 (0.01) | 0.07 (0.01) | 0.07 (0.01) |
|  | RMM3 | 0.07 (0.01) | 0.07 (0.01) | 0.07 (0.01) | 0.07 (0.01) |
| $h_{m_{SB}}^{2}$ | SMM | 0.11 (0.01) | 0.11 (0.01) | 0.11 (0.01) | 0.11 (0.01) |
|  | RMM1 | 0.10 (0.01) | 0.10 (0.01) | 0.10 (0.01) | 0.10 (0.01) |
|  | RMM2 | 0.10 (0.07) | 0.09 (0.01) | 0.09 (0.01) | 0.09 (0.01) |
|  | RMM3 | 0.08 (0.01)) | 0.08 (0.01) | 0.08 (0.01) | 0.08 (0.01) |

^a^ SMM: standard mixed model; RMM1: Recursive mixed model assuming that the relationship between residuals is due to the recursive relationships between the gestation length (GL) phenotype and the liabilities to calving difficulty (CD) and stillbirth (SB); RMM2: RMM assuming that the relationships both between residuals and between herd-years are due to the recursive relationships between the phenotype of GL and the liabilities to CD and SB; RMM3: recursive mixed model assuming that phenotypic correlations of the system are uniquely caused by the recursiveness

**Table 2 - Posterior means (standard deviations) of the genetic correlations, for gestations within 261-267 d**

|  | Model ^a^ | d_CD_ ^b^ | d_SB_ | m_GL_ ^c^ | m_CD_ | m_SB_ |
| --- | --- | --- | --- | --- | --- | --- |
| d_GL_ | SMM | 0.29 (0.06)** | 0.16 (0.08)* | -0.19 (0.07)** | 0.04 (0.08) | 0.03 (0.08) |
|  | RMM1 | 0.13 (0.08) | -0.30 (0.10)** | -0.20 (0.07) | 0.05 (0.05) | 0.04 (0.04) |
|  | RMM2 | 0.13 (0.07) | -0.30 (0.10)** | -0.20 (0.07)** | 0.05 (0.05) | 0.04 (0.04) |
|  | RMM3 | 0.04 (0.04) | -0.45 (0.06)** | -0.20 (0.07)** | 0.00 (0.00) | 0.04 (0.01) |
| d_CD_ | SMM |  | 0.80 (0.04)** | 0.08 (0.10) | -0.04 (0.11) | -0.07 (0.10) |
|  | RMM1 |  | 0.62 (0.07)** | 0.15 (0.18) | -0.04 (0.10) | -0.06 (0.09) |
|  | RMM2 |  | 0.62 (0.07)** | 0.15 (0.18) | -0.04 (0.10) | -0.05 (0.09) |
|  | RMM3 |  | 0.46 (0.05)** | -0.01 (0.01) | -0.01 (0.04) | -0.01 (0.04) |
| d_SB_ | SMM |  |  | 0.05 (0.10) | -0.01 (0.01) | -0.14 (0.10) |
|  | RMM1 |  |  | 0.22 (0.24) | -0.01 (0.01) | -0.19 (0.11) |
|  | RMM2 |  |  | 0.22 (0.24) | -0.01 (0.01) | -0.18 (0.11) |
|  | RMM3 |  |  | 0.09 (0.03)** | -0.01 (0.00) | -0.16 (0.09) † |
| m_GL_ | SMM |  |  |  | 0.18 (0.09)* | -0.15 (0.08)† |
|  | RMM1 |  |  |  | 0.12 (0.09) | -0.30 (0.09)** |
|  | RMM2 |  |  |  | 0.12 (0.09) | -0.31 (0.09)** |
|  | RMM3 |  |  |  | 0.02 (0.02) | -0.17 (0.03)** |
| m_CD_ | SMM |  |  |  |  | 0.70 (0.05)** |
|  | RMM1 |  |  |  |  | 0.56 (0.07)** |
|  | RMM2 |  |  |  |  | 0.56 (0.07)** |
|  | RMM3 |  |  |  |  | 0.39 (0.04)** |

** 99% highest posterior density region, HPD_99%_, does not include 0; * HPD_95%_ does not include 0; † HPD_90%_ does not include 0; ^a^ RMM1: Recursive mixed model assuming that the relationship between residuals is due to the recursive relationships between the gestation length (GL) phenotype and the liabilities to calving difficulty (CD) and stillbirth (SB); RMM2: RMM assuming that the relationships both between residuals and between herd-years are due to the recursive relationships between the phenotype of GL and the liabilities to CD and SB; RMM3: recursive mixed model assuming that phenotypic correlations of the system are uniquely caused by the recursiveness; ^b^ d: direct effect; ^c^ m: maternal effect

**Table 3 - Posterior means (standard deviations) of the genetic correlations, for gestations within 268-273 d**

|  | Model ^a^ | d_CD_ ^b^ | d_SB_ | m_GL_ ^c^ | m_CD_ | m_SB_ |
| --- | --- | --- | --- | --- | --- | --- |
| d_GL_ | SMM | 0.29 (0.06)** | 0.16 (0.08)* | -0.19 (0.07)** | 0.04 (0.08) | 0.03 (0.08) |
|  | RMM1 | 0.23 (0.07)** | -0.07 (0.09) | -0.20 (0.07)** | 0.04 (0.05) | 0.02 (0.04) |
|  | RMM2 | 0.23 (0.06)** | -0.07 (0.09) | -0.20 (0.07)** | 0.04 (0.05) | 0.03 (0.04) |
|  | RMM3 | 0.15 (0.02)** | -0.23 (0.04)** | -0.20 (0.07)** | -0.01 (0.01) | 0.02 (0.01) |
| d_CD_ | SMM |  | 0.80 (0.04)** | 0.08 (0.10) | -0.04 (0.11) | -0.07 (0.10) |
|  | RMM1 |  | 0.65 (0.07)** | 0.13 (0.18) | -0.02 (0.10) | -0.04 (0.09) |
|  | RMM2 |  | 0.65 (0.06)** | 0.13 (0.18) | -0.03 (0.10) | -0.03 (0.09) |
|  | RMM3 |  | 0.48 (0.04)** | -0.03 (0.01)** | -0.04 (0.10) | -0.01 (0.04) |
| d_SB_ | SMM |  |  | 0.05 (0.10) | -0.01 (0.01) | -0.14 (0.10) |
|  | RMM1 |  |  | 0.18 (0.25) | -0.01 (0.01) | -0.18 (0.11) |
|  | RMM2 |  |  | 0.19 (0.25) | -0.01 (0.01) | -0.16 (0.12) |
|  | RMM3 |  |  | 0.05 (0.02)** | 0.00 (0.00) | 0.17 (0.10) |
| m_GL_ | SMM |  |  |  | 0.18 (0.09)* | -0.15 (0.08)† |
|  | RMM1 |  |  |  | 0.16 (0.09)† | -0.23 (0.09)** |
|  | RMM2 |  |  |  | 0.17 (0.09)† | -0.23 (0.09)** |
|  | RMM3 |  |  |  | 0.07 (0.01)** | -0.08 (0.02)** |
| m_CD_ | SMM |  |  |  |  | 0.70 (0.05)** |
|  | RMM1 |  |  |  |  | 0.56 (0.07)** |
| l | RMM2 |  |  |  |  | 0.56 (0.06)** |
|  | RMM3 |  |  |  |  | 0.38 (0.03)** |

** 99% highest posterior density region, HPD_99%_, does not include 0; * HPD_95%_ does not include 0; † HPD_90%_ does not include 0; ^a^ RMM1: Recursive mixed model assuming that the relationship between residuals is due to the recursive relationships between the gestation length (GL) phenotype and the liabilities to calving difficulty (CD) and stillbirth (SB); RMM2: RMM assuming that the relationships both between residuals and between herd-years are due to the recursive relationships between the phenotype of GL and the liabilities to CD and SB; RMM3: recursive mixed model assuming that phenotypic correlations of the system are uniquely caused by the recursiveness; ^b^ d: direct effect; ^c^ m: maternal effect

**Table 4 - Posterior means (standard deviations) of the genetic correlations, for gestations within 274-279 d**

|  | Model ^a^ | d_CD_ ^b^ | d_SB_ | m_GL_ ^c^ | m_CD_ | m_SB_ |
| --- | --- | --- | --- | --- | --- | --- |
| d_GL_ | SMM | 0.29 (0.06)** | 0.16 (0.08)* | -0.19 (0.07)** | 0.04 (0.08) | 0.03 (0.08) |
|  | RMM1 | 0.31 (0.07)** | 0.06 (0.10) | -0.20 (0.07)** | 0.03 (0.05) | 0.01 (0.04) |
|  | RMM2 | 0.30 (0.07)** | 0.06 (0.10) | -0.20 (0.07)** | 0.03 (0.05) | 0.02 (0.04) |
|  | RMM3 | 0.22 (0.04)** | -0.09 (0.07) | -0.20 (0.07) | -0.02 (0.01) | 0.01 (0.01) |
| d_CD_ | SMM |  | 0.80 (0.04)** | 0.08 (0.10) | -0.04 (0.11) | -0.07 (0.10) |
|  | RMM1 |  | 0.67 (0.07)** | 0.11 (0.18) | -0.02 (0.10) | -0.04 (0.09) |
|  | RMM2 |  | 0.67 (0.06)** | 0.11 (0.18) | -0.02 (0.10) | -0.02 (0.09) |
|  | RMM3 |  | 0.49 (0.05)** | -0.05 (0.02)** | -0.04 (0.10) | -0.01 (0.04) |
| d_SB_ | SMM |  |  | 0.05 (0.10) | -0.01 (0.01) | -0.14 (0.10) |
|  | RMM1 |  |  | 0.16 (0.25) | -0.01 (0.01) | -0.17 (0.11) |
|  | RMM2 |  |  | 0.16 (0.25) | -0.01 (0.01) | -0.16 (0.12) |
|  | RMM3 |  |  | 0.02 (0.02) | 0.00 (0.00) | -0.17 (0.10) |
| m_GL_ | SMM |  |  |  | 0.18 (0.09)* | -0.15 (0.08) † |
|  | RMM1 |  |  |  | 0.20 (0.09)* | -0.18 (0.09)* |
|  | RMM2 |  |  |  | 0.20 (0.09)* | -0.19 (0.09)* |
|  | RMM3 |  |  |  | 0.11 (0.02)** | -0.03 (0.02) |
| m_CD_ | SMM |  |  |  |  | 0.70 (0.05)** |
|  | RMM1 |  |  |  |  | 0.56 (0.07)** |
|  | RMM2 |  |  |  |  | 0.56 (0.07)** |
|  | RMM3 |  |  |  |  | 0.38 (0.03)** |

** 99% highest posterior density region, HPD_99%_, does not include 0; * HPD_95%_ region does not include 0; † HPD_90%_ region does not include 0; ^a^ RMM1: Recursive mixed model assuming that the relationship between residuals is due to the recursive relationships between the gestation length (GL) phenotype and the liabilities to calving difficulty (CD) and stillbirth (SB); RMM2: RMM assuming that the relationships both between residuals and between herd-years are due to the recursive relationships between the phenotype of GL and the liabilities to CD and SB; RMM3: recursive mixed model assuming that phenotypic correlations of the system are uniquely caused by the recursiveness; ^b^ d: direct effect; ^c^ m: maternal effect

**Table 5 - Posterior means (standard deviations) of the genetic correlations, for gestations within 280-291 d**

|  | Model ^a^ | d_CD_ ^b^ | d_SB_ | m_GL_ ^c^ | m_CD_ | m_SB_ |
| --- | --- | --- | --- | --- | --- | --- |
| d_GL_ | SMM | 0.29 (0.06)** | 0.16 (0.08)* | -0.19 (0.07)** | 0.04 (0.08) | 0.03 (0.08) |
|  | RMM1 | 0.36 (0.06)** | 0.37 (0.08)** | -0.20 (0.07)** | 0.02 (0.05) | -0.01 (0.04) |
|  | RMM2 | 0.35 (0.06)** | 0.38 (0.08)** | -0.20 (0.07)** | 0.02 (0.05) | -0.01 (0.04) |
|  | RMM3 | 0.28 (0.02)** | 0.28 (0.04)** | -0.20 (0.07)** | -0.03 (0.01)** | -0.02 (0.01)** |
| d_CD_ | SMM |  | 0.80 (0.04)** | 0.08 (0.10) | -0.04 (0.11) | -0.07 (0.10) |
|  | RMM1 |  | 0.73 (0.05)** | 0.10 (0.17) | -0.01 (0.10) | -0.03 (0.09) |
|  | RMM2 |  | 0.73 (0.05)** | 0.09 (0.17) | -0.02 (0.10) | -0.01 (0.09) |
|  | RMM3 |  | 0.57 (0.04)** | -0.06 (0.02)** | -0.04 (0.10) | -0.02 (0.04) |
| d_SB_ | SMM |  |  | 0.05 (0.10) | -0.01 (0.01) | -0.14 (0.10) |
|  | RMM1 |  |  | 0.08 (0.23) | -0.01 (0.01) | -0.15 (0.11) |
|  | RMM2 |  |  | 0.08 (0.23) | -0.01 (0.01) | -0.13 (0.11) |
|  | RMM3 |  |  | -0.06 (0.02)** | 0.00 (0.00) | -0.17 (0.10)† |
| m_GL_ | SMM |  |  |  | 0.18 (0.09)* | -0.15 (0.08) † |
|  | RMM1 |  |  |  | 0.23 (0.09)** | -0.07 (0.09) |
|  | RMM2 |  |  |  | 0.23 (0.09)** | -0.08 (0.09) |
|  | RMM3 |  |  |  | 0.13 (0.01)** | 0.10 (0.01)** |
| m_CD_ | SMM |  |  |  |  | 0.70 (0.05)** |
|  | RMM1 |  |  |  |  | 0.59 (0.07)** |
|  | RMM2 |  |  |  |  | 0.59 (0.06)** |
|  | RMM3 |  |  |  |  | 0.40 (0.03)** |

** 99% highest posterior density region, HPD_99%_, does not include 0; * HPD_95%_ does not include 0; † HPD_90%_ does not include 0; ^a^ RMM1: Recursive mixed model assuming that the relationship between residuals is due to the recursive relationships between the gestation length (GL) phenotype and the liabilities to calving difficulty (CD) and stillbirth (SB); RMM2: RMM assuming that the relationships both between residuals and between herd-years are due to the recursive relationships between the phenotype of GL and the liabilities to CD and SB; RMM3: recursive mixed model assuming that phenotypic correlations of the system are uniquely caused by the recursiveness; ^b^ d: direct effect; ^c^ m: maternal effect

**Table 6 - Posterior means (standard deviations) of correlations between contemporary (h) groups and residual (e) effects**

|  | Model ^a^ | Category of GL | | | |
| --- | --- | --- | --- | --- | --- |
|  |  | 261-267 d | 268-273 d | 274-279 d | 280-291 d |
| $\rho_{h_{GL}h_{CD}}$ | SMM | 0.04 (0.04) | 0.04 (0.04) | 0.04 (0.04) | 0.04 (0.04) |
|  | RMM1 | 0.01 (0.04) | 0.03 (0.04) | 0.05 (0.04) | 0.06 (0.04) |
|  | RMM2 | 0.01 (0.01) | 0.03 (0.00)** | 0.04 (0.01)** | 0.05 (0.00)** |
|  | RMM3 | 0.01 (0.01) | 0.03 (0.00)** | 0.04 (0.01)** | 0.06 (0.00)** |
| $\rho_{h_{GL}h_{SB}}$ | SMM | 0.13 (0.05)** | 0.13 (0.05)** | 0.13 (0.05)** | 0.13 (0.05)** |
|  | RMM1 | 0.00 (0.05) | 0.09 (0.05) † | 0.13 (0.05)* | 0.24 (0.05)** |
|  | RMM2 | -0.10 (0.01)** | -0.05 (0.01)** | -0.02 (0.01) | 0.06 (0.01)** |
|  | RMM3 | -0.10 (0.01)** | -0.05 (0.01)** | -0.01 (0.01) | 0.06 (0.01)** |
| $\rho_{h_{CD}h_{SB}}$ | SMM | 0.04 (0.05) | 0.04 (0.05) | 0.04 (0.05) | 0.04 (0.05) |
|  | RMM1 | 0.02 (0.08) | 0.00 (0.05) | 0.00 (0.05) | 0.01 (0.05) |
|  | RMM2 | 0.54 (0.03)** | 0.54 (0.02)** | 0.53 (0.02)** | 0.54 (0.02)** |
|  | RMM3 | 0.54 (0.03)** | 0.54 (0.02)** | 0.54 (0.02)** | 0.54 (0.02)** |
| $\rho_{e_{GL}e_{CD}}$ | SMM | 0.10 (0.01)** | 0.10 (0.01)** | 0.10 (0.01)** | 0.10 (0.01)** |
|  | RMM1 | 0.02 (0.02) | 0.07 (0.01)** | 0.10 (0.02)** | 0.13 (0.01)** |
|  | RMM2 | 0.02 (0.02) | 0.07 (0.01)** | 0.10 (0.02)** | 0.13 (0.01)** |
|  | RMM3 | 0.02 (0.02) | 0.07 (0.01)** | 0.11 (0.02)** | 0.13 (0.01)** |
| $\rho_{e_{GL}e_{SB}}$ | SMM | 0.00 (0.00) | 0.00 (0.00) | 0.00 (0.00) | 0.00 (0.00) |
|  | RMM1 | -0.17 (0.02)** | -0.08 (0.02)** | -0.03 (0.02) | 0.10 (0.01)** |
|  | RMM2 | -0.17 (0.02)** | -0.08 (0.02)** | -0.03 (0.02) | 0.10 (0.01)** |
|  | RMM3 | -0.17 (0.02)** | -0.08 (0.01)** | -0.03 (0.02) | 0.10 (0.01)** |
| $\rho_{e_{CD}e_{SB}}$ | SMM | 0.09 (0.01)** | 0.09 (0.01)** | 0.09 (0.01)** | 0.09 (0.01)** |
|  | RMM1 | 0.40 (0.02)** | 0.40 (0.01)** | 0.40 (0.01)** | 0.41 (0.01)** |
|  | RMM2 | 0.39 (0.02)** | 0.38 (0.01)** | 0.38 (0.01)** | 0.40 (0.01)** |
|  | RMM3 | 0.39 (0.02)** | 0.39 (0.01)** | 0.39 (0.01)** | 0.40 (0.01)** |

** 99% highest posterior density, HPD_99%_, region does not include 0; * HPD_95%_ does not include 0; † HPD_90%_ does not include 0; ^a^ RMM1: Recursive mixed model assuming that the relationship between residuals is due to the recursive relationships between the gestation length (GL) phenotype and the liabilities to calving difficulty (CD) and stillbirth (SB); RMM2: RMM assuming that the relationships both between residuals and between herd-years are due to the recursive relationships between the phenotype of GL and the liabilities to CD and SB; RMM3: recursive mixed model assuming that phenotypic correlations of the system are uniquely caused by the recursiveness
